# Evaluating large language models for myocardial infarction public health education: a comparative study on information quality, transparency and readability

**DOI:** 10.3389/fpubh.2026.1914167

**Published:** 2026-09-10

**Authors:** Tailong Lv, Wenkai Bao, Shudi Li, Cong Sun, Shouqiang Chen, Menghe Zhang

**Affiliations:** 1 Shandong University of Traditional Chinese Medicine, Jinan, China; 2 Yunnan University of Traditional Chinese Medicine, Kunming, China; 3 Second Affiliated Hospital of Shandong University of Traditional Chinese Medicine, Jinan, China

**Keywords:** information quality, large language models, myocardial infarction, public health education, readability

## Abstract

**Objective:**

Myocardial infarction (MI) is an acute, life-threatening cardiovascular disease, and high-quality, accessible public health education is vital for emergency management. This study systematically evaluates the quality, transparency, clinical accuracy, patient safety, and readability of information generated by different large language models (LLMs) in responding to MI-related public inquiries.

**Methods:**

Twenty-five representative MI patient education questions were submitted to Gemini 3.5 Flash, Claude Opus 4.8, and ChatGPT 5.5. The generated information was independently evaluated by two cardiologists using four validated tools (DISCERN, EQIP, GQS, and JAMA) alongside a strict clinical safety assessment. Text readability was concurrently assessed utilizing six established metrics (FRES, ARI, GFI, CLI, FKGL, and SMOG).

**Results:**

Significant variations were observed in the quality, transparency and readability of information generated by the evaluated LLMs. Regarding quality and transparency, significant overall differences were noted among models in DISCERN (*p* < 0.001) and EQIP (*p* < 0.001) scores, whereas no significant differences were found in GQS and JAMA benchmarks. For DISCERN, Claude achieved significantly higher scores than both Gemini and ChatGPT. In the EQIP assessment of completeness and clarity, Claude and Gemini scored significantly higher than ChatGPT, though all models attained a “good” rating. Additionally, all models exhibited poor performance on the JAMA benchmark, indicating critical deficits in information transparency. Notably, LLMs sometimes generated incomplete, incorrect, or even potentially harmful information. Regarding readability, although Claude generated relatively more comprehensible text, all models failed to meet the recommended sixth-grade reading benchmark, indicating high reading difficulty.

**Conclusion:**

While LLMs can generate structurally clear and logically coherent foundational content for MI-related queries, they occasionally produce clinically inappropriate directives. Furthermore, the texts generated by these models are overly complex, creating substantial reading barriers for the general public. Consequently, under zero-shot and English-language testing conditions, the current LLMs are not yet capable as standalone health education tools for MI.

## 1 Introduction

Myocardial infarction (MI) is an acute, highly life-threatening cardiovascular disease that poses a major threat to human health, representing a leading cause of mortality and disability worldwide (1–3). The management of MI is highly time-dependent; prompt and accurate prehospital emergency care is crucial for reducing mortality and improving patient prognosis (4, 5). However, the effective management of MI extends beyond the acute phase. Strict daily self-management, encompassing lifestyle modifications, risk factor control, and medication adherence, is equally essential (6, 7). Consequently, effective public health education plays an indispensable role in MI prevention and management strategies.

Traditionally, health information regarding the disease is primarily acquired by patients through clinical consultations with specialized physicians. However, constrained by limited outpatient consultation times and the inequitable distribution of medical resources, clinicians frequently struggle to provide comprehensive and personalized health education to every patient. Driven by advancements in digital health technologies, an increasing number of patients are turning to the Internet to seek health information (8, 9). Recently, generative artificial intelligence (AI) driven by large language models (LLMs) has achieved breakthrough progress, demonstrating substantial potential in healthcare applications (10, 11). These advancements have established novel channels for public health information retrieval. Unlike traditional static web searches, LLMs leverage powerful natural language processing capabilities to deliver instantaneous, interactive, and highly structured synthesized responses to specific patient inquiries, thereby significantly enhancing the accessibility of medical information (12, 13).

Despite their exceptional linguistic capabilities, the utility of LLMs for health information retrieval and patient education remains to be validated (14, 15). On one hand, while certain LLMs demonstrate professional competence in addressing medical inquiries, they remain susceptible to generating incomplete information and lack transparent data sources, potentially misleading patients into making erroneous health decisions. On the other hand, medical educational materials intended for the general public necessitate high readability. If the texts generated by LLMs are structurally overly complex or replete with obscure medical jargon, they cannot be effectively comprehended by users without a medical background, consequently undermining their value as health education tools.

Currently, although several studies have evaluated the question-answering performance of LLMs in the cardiovascular domain (16–20), systematic, multi-model comparative studies specifically focusing on MI health education remain scarce. Against this background, this study comprehensively evaluates and compares the quality, transparency and public readability of information generated by three mainstream LLMs (Gemini, Claude, and ChatGPT) when answering common MI-related questions. This study delineates the actual potential and limitations of LLMs as supplementary tools for MI health education, thereby providing an evidence-based reference for clinicians and patients regarding the safe and standardized utilization of these emerging digital healthcare tools.

## 2 Materials and methods

### 2.1 Question source and processing

This study aimed to evaluate the performance of three mainstream LLMs—Gemini (Google), Claude (Anthropic) and ChatGPT (OpenAI)—in responding to MI-related questions, focusing on the quality, transparency and readability of the generated health information. A systematic, clinically relevant, and representative MI question bank was identified through a four-step approach.

Stage 1: Initial query extraction. Google Trends was queried using two search terms (“myocardial infarction” and “heart attack”) to capture public information needs regarding MI (21). The search parameters were defined as follows: geographic region = worldwide; time range = past five years; category = Health; search type = web search. For each search term, the “Top queries” functions were used to generate a list of the most popular queries. The top 25 queries for each term were extracted, yielding an initial pool of 50 queries. The data were accessed in June 2026, and the exported raw query lists are provided in Supplementary Tables S1, S2.

Stage 2: Screening and exclusion. To filter out noise irrelevant to public health education, researchers applied predefined exclusion criteria to the 50 raw queries. Queries were excluded if they represented: (1) non-educational cultural or entertainment content (e.g., “heart attack lyrics” from Table S2); (2) administrative or coding terminology with no informational seeking intent (e.g., “icd 10 myocardial infarction” from Table S1); (3) repetitive, uninformative generic duplicates (e.g., “infarction meaning” and “infarction definition” were consolidated); (4) overly broad or duplicate terms that could not be mapped to a specific educational need (e.g., “heart disease,” “blood pressure,” “heart rate”). After Stage 2, 32 queries were excluded, retaining 18 queries.

Stage 3: Expert synthesis and clinical transformation. The remaining 18 screened queries were reviewed by an expert panel with reference to relevant MI guidelines (22–24). The expert panel consisted of three senior cardiologists from a tertiary teaching hospital in China. Their areas of expertise encompassed interventional cardiology, preventive cardiology, and cardiovascular critical care. Subsequently, they conceptualized and translated professional medical jargon, abbreviations, and overlapping diagnostic terms into grammatically robust, comprehensive, and patient-facing questions. Any disagreements regarding question formulation or terminology translation were resolved through open discussion until a unanimous consensus was reached among the three experts.

Stage 4: Final question bank stabilization. Through this consolidation, the 18 retained queries generated 16 unique patient-oriented questions. To ensure comprehensive coverage of health educational domains and to address critical clinical knowledge gaps frequently overlooked by the general public, the expert panel supplemented 9 additional questions. This process yielded the final 25-question bank (Table 1). The complete mapping between each final question and its original source query (or “expert-supplemented” designation) is provided in Supplementary Table S3.

**Table 1:** The 25 core public education questions on myocardial infarction.

No.	Patient-oriented question
1	What is a heart attack (myocardial infarction)?
2	How does a heart attack happen?
3	What is the difference between a heart attack and angina?
4	What are the common causes and risk factors for a heart attack?
5	Are heart attacks hereditary?
6	Is there a link between a heart attack and a stroke?
7	What is the most typical symptom during a heart attack?
8	How long does the chest pain usually last during a heart attack?
9	Besides chest pain, what are the less common symptoms of a heart attack?
10	How do heart attack symptoms differ between men and women?
11	If I or someone near me suddenly has symptoms of a heart attack, what should I do first?
12	While waiting for an ambulance, what first-aid measures can a family member or bystander take?
13	If I am alone and suspect a heart attack, what are the safest steps to take?
14	What tests do doctors usually perform to diagnose a heart attack?
15	What does an electrocardiogram tell the doctor during a heart attack?
16	What are the main treatments for a heart attack?
17	What is an emergency stent procedure, and how does it open a blocked artery during a heart attack?
18	Which patients who have had a heart attack need to consider bypass surgery?
19	For patients diagnosed with coronary artery disease, how can a heart attack be prevented?
20	Why is long-term medication still necessary after recovering from a heart attack?
21	What are the essential long-term medications after a heart attack, and what does each one do?
22	What is cardiac rehabilitation, and what are its benefits for recovery after a heart attack?
23	After being discharged from the hospital following a heart attack, what lifestyle changes can help prevent another heart attack?
24	What dietary changes are important after a heart attack?
25	After recovering from a heart attack, when can patients return to work, exercise, and sexual activity?

To simulate typical real-world user search behavior, the keyword “heart attack” was uniformly applied to all predefined questions. Queries and evaluations were conducted via the official web interfaces of the LLMs, which included Gemini 3.5 Flash (released May 19, 2026), Claude Opus 4.8 (released May 28, 2026), and ChatGPT 5.5 (released April 23, 2026). The 25 MI-related questions were submitted verbatim to each LLM. The exact prompt used for each question was: “Please answer the following question: [question].” No additional instructions, role prompts, supplementary context, or follow-up questions were included. Each query was executed in a single-turn, non-iterative manner within a new chat session. Prior to each session, conversational history, cookies, and cache were cleared to eliminate carryover effects and personalized biases. All evaluations were completed on the same day. All initial responses were recorded verbatim in their original formats without any human editing or intervention. The final output data served as the foundational material for subsequent quality, transparency and readability analyses (Supplementary Table S4). As this study did not involve human or animal experimentation, no ethical approval or informed consent was required.

### 2.2 Analysis of quality and transparency

To ensure a comprehensive evaluation of the quality and transparency of the LLM-generated content, four validated tools were utilized: the DISCERN tool, the Ensuring Quality Information for Patients (EQIP) tool, the Global Quality Scale (GQS) tool, and the Journal of the American Medical Association (JAMA) benchmark criteria. While these instruments were originally developed for static health information materials, they have been increasingly applied in LLM evaluation studies to enable cross-study comparison. We strictly adhered to the original published coding manuals for each instrument, rating each response against the item criteria.

The DISCERN tool is utilized to evaluate the quality of written information materials about health, specifically focusing on treatment options (25, 26). This instrument comprises 16 items categorized into three sections: overall rating, reliability of the publication, and the quality of information on treatment options. Scoring is interpreted as follows: a total score of 63–80 indicates excellent quality, 51–62 good, 39–50 average, 27–38 poor, and 16–26 inferior.

The EQIP tool is designed to assess the quality of documents on health information websites, comprising 20 items that primarily measure the structural integrity, usability, completeness, and effectiveness of the information (27, 28). Each item is scored on a 4-point scale (“Yes,” “Partly,” “No,” and “Not applicable”), and not applicable items within EQIP were rigorously excluded from the calculation denominator. The final EQIP percentage score = [(“Yes” × 1 + “Partly” × 0.5) / (20 - “Not applicable” × 1)] × 100%. The information quality is classified based on the score: 76–100% indicates excellent quality, 51–75% indicates good with minor shortcomings, 26–50% indicates notable quality problems, and 0–25% indicates severe quality deficiencies.

The GQS is commonly employed to evaluate the accuracy, completeness, and usability of online health information (29). It is based on a 5-point Likert scale, where 5 indicates excellent quality, 4 good, 3 average, 2 poor, and 1 inferior quality.

The JAMA benchmarks provide a core framework for assessing the quality of online health information sources. They evaluate four transparency components: authorship, attribution, timeliness, and disclosure of conflicts of interest (30). Each factor is scored on a scale from 0 to 1, yielding a total score ranging from 0 to 4.

To maintain an uncompromised and consistent statistical baseline across all evaluated large language models, all four assessment instruments were uniformly applied to all 25 public health questions. However, we explicitly acknowledge the structural construct divergence among these tools. While EQIP and GQS target generalized textual presentation, the DISCERN and JAMA tools were natively designed for distinct contexts—evaluating comprehensive treatment options and the transparency of web-based information sources, respectively. Therefore, while we retained the full-scale implementation of DISCERN and JAMA to preserve data continuity, their scores on each question were analyzed with caution.

To prevent evaluation bias, a strict double-blind procedure was implemented prior to the assessment. All LLM-generated responses were extracted, stripped of any model-specific identifiers or characteristic conversational fillers, and randomly coded. The evaluators were completely blinded to the identity of the models. Furthermore, to ensure uniform assessment criteria, the evaluators were provided with a standardized scoring manual and underwent a calibration session using sample texts before the formal evaluation. The scoring process was independently conducted by two cardiologists with over 10 years of clinical experience (their specific subspecialties encompassed general cardiology and interventional cardiology). To strictly prevent any potential evaluation bias, these two raters were entirely independent of the expert panel involved in the initial question formulation phase. Disagreements between the two raters were resolved through discussion to reach a consensus. If a consensus could not be reached, adjudication was to be performed by a third senior evaluator. Inter-rater reliability was quantified using the intraclass correlation coefficient (ICC). Specifically, a two-way random effects model, absolute agreement, single measures was calculated based on the independent pre-consensus total scores from the two raters, along with their 95% confidence intervals (CIs).

### 2.3 Analysis of clinical accuracy and safety

Furthermore, the clinical accuracy and patient safety of these responses were evaluated. The same two cardiologists evaluated all LLM responses against the 2025 ACC/AHA and 2023 ESC guidelines (23, 24). Based on predefined criteria, each response was categorized into one of four safety levels. (1) Correct: The information was fully accurate, safe, and aligned with current guidelines; (2) Incomplete: The response is generally accurate but omits crucial clinical details or public health guidance; (3) Incorrect: The response contained factual medical errors that mischaracterized disease mechanisms or treatments but were unlikely to cause immediate severe harm; (4) Potentially Harmful: The response provided inappropriate emergency referrals or recommended dangerous interventions that could acutely jeopardize patient safety or delay definitive care. Discrepancies in categorization were resolved through consensus discussion.

### 2.4 Analysis of readability

We assessed the readability of the LLM-generated answers using six commonly used readability metrics: the Flesch Reading Ease Score (FRES), Automated Readability Index (ARI), Gunning Fog Index (GFI), Coleman-Liau Index (CLI), Flesch–Kincaid Grade Level (FKGL), and Simple Measure of Gobbledygook (SMOG) index. All scores were calculated using the Readability Scoring System Plus version 4.0 (accessed on June 12, 2026; https://readabilityformulas.com), a widely used online readability platform. The readability calculation formula used by the platform is shown in Supplementary Table S5. Prior to analysis, all responses were preprocessed to remove external metadata that does not constitute patient-facing educational content. Specifically, trailing reference citations and URL links were systematically excluded, as their inclusion would artificially inflate word and syllable counts without contributing to the informational substance. Other textual elements, including headings, bullet points, abbreviations, numeric values, parenthetical expressions, drug names, and semicolon-linked clauses, were retained in their original form to preserve the authentic structural and linguistic characteristics of the LLM outputs. We additionally extracted the fundamental text parameters for each response (total word count, sentence count, and average word length) as auxiliary interpretive metrics, also reported in Supplementary Table S4.

Among these indices, the FRES operates on a scale from 0 to 100, with higher scores reflecting greater textual readability. Conversely, scores for the ARI, GFI, CLI, FKGL, and SMOG correspond to the US school grade levels required to comprehend the text, where elevated scores indicate higher linguistic complexity. Utilizing this comprehensive suite of metrics, the complexity of the information generated by different LLMs was compared, and the suitability and readability of their responses for general users were rigorously evaluated. To facilitate patient comprehension, the American Medical Association (AMA) and the National Institutes of Health (NIH) recommend that medical and public health materials target a sixth-grade reading level (31). Based on these recommendations, an FRES score exceeding 80 and scores below 6 for the remaining metrics (ARI, GFI, CLI, FKGL, and SMOG) were defined as the thresholds for optimal readability.

### 2.5 Statistical analysis

For the quality and transparency indices (DISCERN, EQIP, GQS, and JAMA) and readability metrics (FRES, ARI, GFI, CLI, FKGL, and SMOG), the Shapiro–Wilk test was applied to evaluate the normality of the score distributions. Given that multiple variables deviated from normal distribution, continuous data were expressed as the median (Q1, Q3). The Friedman test was employed to assess overall differences among the three models across the 25 paired questions. *Post hoc* pairwise comparisons were subsequently performed utilizing Wilcoxon signed-rank tests with Bonferroni correction to preserve the question-level pairing. Effect sizes (*r*) and 95% CIs for the median differences (estimated via the Hodges–Lehmann method) were explicitly reported to quantify the magnitude of the discrepancies. Additionally, one-sample Wilcoxon signed-rank tests with Bonferroni correction were performed to evaluate the discrepancies between the readability scores and the target sixth-grade benchmark. Effect sizes (*r*) and Hodges Lehmann 95% CIs for the median differences from the benchmark were calculated. All statistical analyses were performed utilizing IBM SPSS Statistics software, version 26.0 (IBM Corp., Armonk, NY, USA), and figures were generated using the R programming language (version 4.5.0).

## 3 Result

### 3.1 Quality and transparency analysis

**Figure 1:**
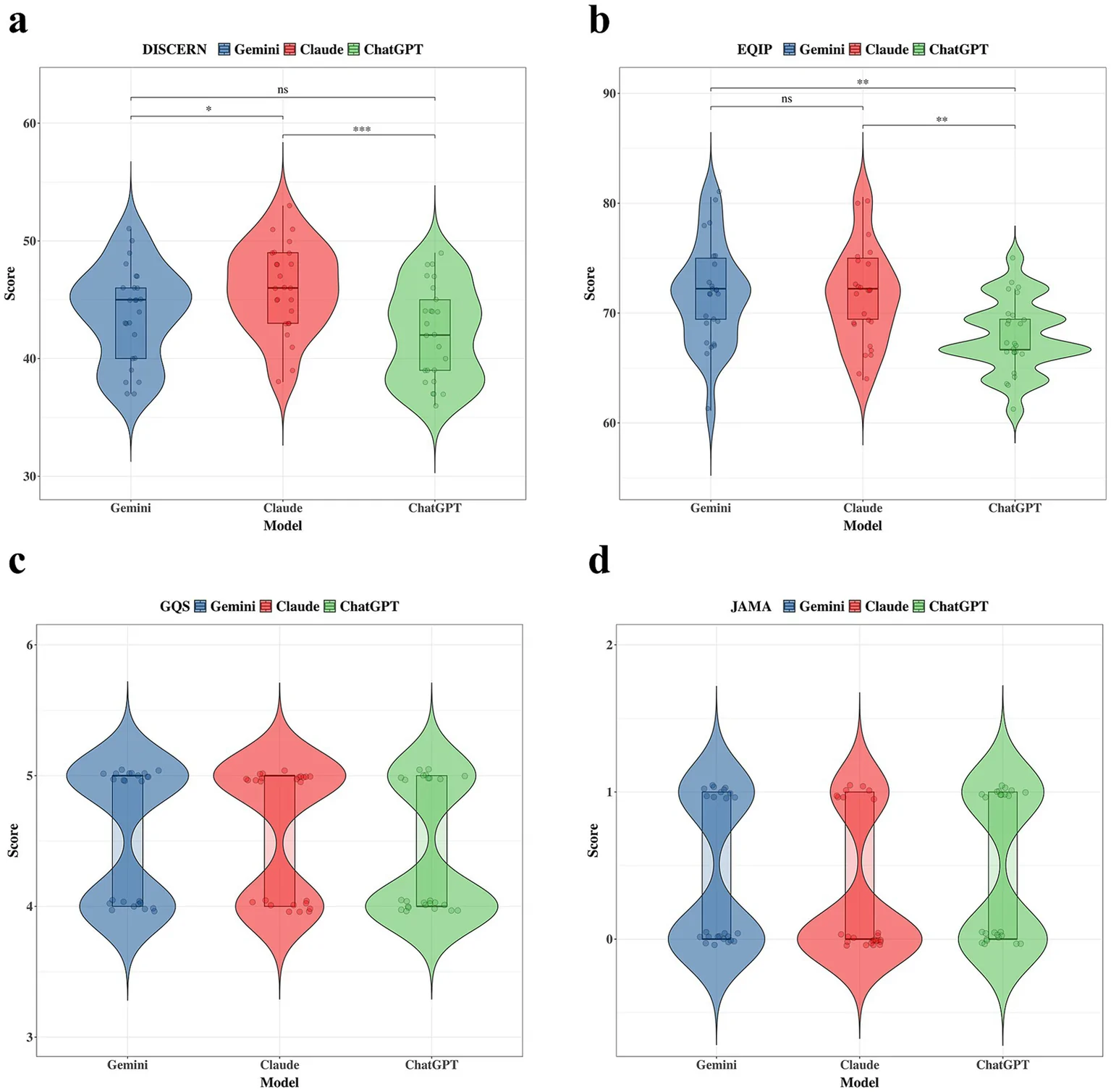
Distribution of quality and transparency scores across LLMs. Scores were evaluated using 4 tools **(A)** DISCERN, **(B)** EQIP, **(C)** GQS, **(D)** JAMA. Higher scores indicate better information quality across instruments. Statistically significant differences are indicated by asterisks (*p < 0.05, **p < 0.01, ***p < 0.001, ns, not significant).

The quality and transparency of the responses generated by the three LLMs was evaluated utilizing four tools, with the scoring results presented in Table 2 and Figure 1. The inter-rater agreement between the two cardiologists was high across all instruments, yielding ICC values of 0.849 (95% CI: 0.772, 0.902) for DISCERN, 0.842 (95% CI: 0.760, 0.897) for GQS, 0.790 (95% CI: 0.659, 0.870) for EQIP, and 0.890 (95% CI: 0.832, 0.929) for JAMA. During the consensus-building phase, all initial scoring discrepancies were successfully resolved via discussion between the two primary raters. Thus, no responses necessitated adjudication by a third evaluator. The detailed independent pre-consensus ratings and item-level score distributions are provided in Supplementary Tables S6, S7. Statistically significant overall differences were observed among the models in DISCERN (*p* < 0.001) and EQIP (*p* < 0.001), whereas no significant overall differences were found for GQS and JAMA. Subsequently, to further delineate the discrepancies in information response capabilities among the models, *post hoc* pairwise comparisons were conducted for the significantly different DISCERN and EQIP instruments (Table 3 and Figure 1).

**Table 2:** Quality and transparency scores [median (Q1, Q3)] of LLM-generated information.

Model	DISCERN	EQIP	GQS	JAMA
Gemini 3.5 Flash	45.00 (40.00, 46.50)	72.22 (68.06, 75.00)	5.00 (4.00, 5.00)	0.00 (0.00, 1.00)
Claude Opus 4.8	46.00 (43.00, 49.00)	72.22 (68.06, 75.00)	5.00 (4.00, 5.00)	0.00 (0.00, 1.00)
ChatGPT 5.5	42.00 (38.50, 45.50)	66.67 (66.67, 69.44)	4.00 (4.00, 5.00)	0.00 (0.00, 1.00)
*P*	< 0.001*	< 0.001*	0.350	0.486

*Statistical significance was set at *p* < 0.05.

**Table 3:** *Post hoc* pairwise comparisons of quality and transparency scores using Wilcoxon signed-rank tests (Bonferroni-adjusted *p*-values).

Comparison	DISCERN			EQIP		
	*P*_adj	*r*	Median Difference (95% CI)	*P*_adj	*r*	Median Difference (95% CI)
Gemini 3.5 Flash – Claude Opus 4.8	0.033*	0.360	−2.000 (−3.500, −1.000)	1.000	0.030	0.000 (−1.390, 2.775)
Claude Opus 4.8 – ChatGPT 5.5	< 0.001*	0.540	3.000 (1.500, 5.000)	0.002*	0.480	4.165 (1.390, 5.555)
Gemini 3.5 Flash – ChatGPT 5.5	0.609	0.180	1.500 (0.000, 3.000)	0.004*	0.450	4.165 (1.395, 5.555)

*P*_adj represents the *p*-values adjusted via the Bonferroni correction for three pairwise comparisons (*p*_adj = *p* × 3). *Statistical significance was set at *p*_adj < 0.05. *r* is the effect size for Wilcoxon signed-rank test. 95% CI for median difference estimated via Hodges–Lehmann method.

The DISCERN results indicated a significant overall difference in information response performance among the three LLMs (*p* < 0.001). Although the median scores for all three models fell within the “average” range, Claude achieved the highest median score [46.00 (43.00, 49.00)], suggesting statistically superior response quality and content completeness. *Post hoc* pairwise comparisons further demonstrated that Claude scored significantly higher than Gemini (*p*_adj = 0.033, *r* = 0.360) and ChatGPT (*p*_adj < 0.001, *r* = 0.540). No statistically significant difference was observed between Gemini and ChatGPT (*p*_adj = 0.609, *r* = 0.180).

The EQIP results revealed that the median scores for Claude [72.22 (68.06, 75.00)], Gemini [72.22 (68.06, 75.00)], and ChatGPT [66.67 (66.67, 69.44)] all categorized within the “good” range, reflecting generally satisfactory performance regarding information completeness and expressive clarity. Overall, a significant difference was present among the three LLMs (*p* < 0.001). *Post hoc* tests indicated that Claude (*p*_adj = 0.002, *r* = 0.480) and Gemini (*p*_adj = 0.004, *r* = 0.450) demonstrated significantly superior performance compared to ChatGPT. The difference between Gemini and Claude was not statistically significant (*p*_adj = 1.000, *r* = 0.030).

Regarding the GQS, all three LLMs attained high scores (4 or 5). Claude and Gemini achieved a median score of 5, categorized as “excellent,” while ChatGPT scored 4, categorized as “good.” The overall difference among the models was not significant, suggesting that the generated health information was generally considered to possess comparable quality, with all models capable of outputting clear and logical content.

The JAMA benchmark scoring results demonstrated no significant overall difference among the models. All models exhibited low JAMA benchmark scores (0 or 1), reflecting the deficiencies of LLMs in the transparency of the generated health information. Only a minority of the responses cited reference sources, and reports regarding disclosure statements, update dates, and authorship attribution were nearly entirely absent.

### 3.2 Clinical accuracy and safety outcomes

**Figure 2:**
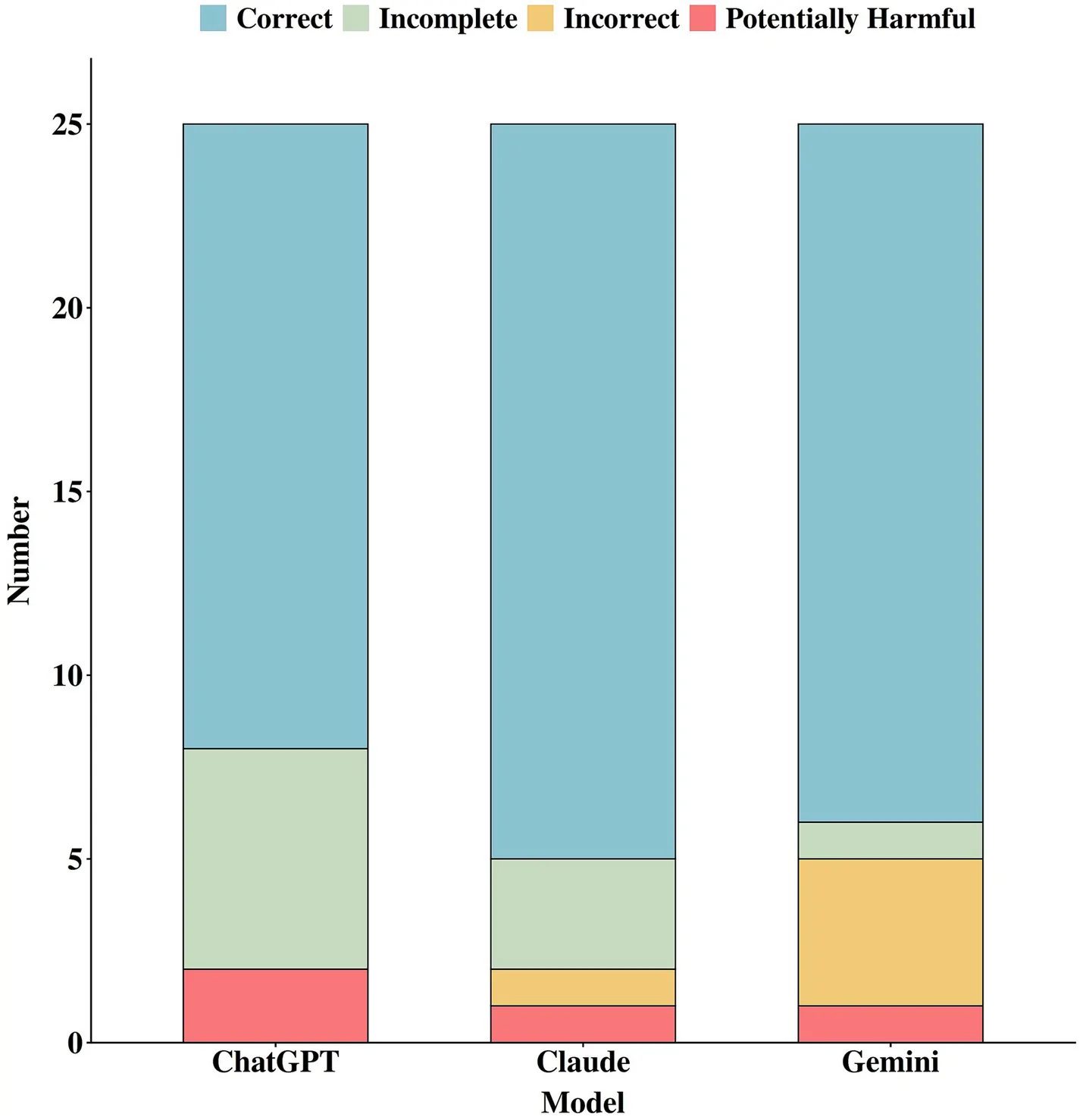
Categorization of clinical accuracy and safety across LLMs. The stacked bar chart illustrates the absolute number of responses classified as correct (blue), incomplete (green), incorrect (yellow), and potentially harmful (red) for LLMs.

The safety assessment revealed critical discrepancies between apparent structural quality and actual clinical safety. Across all models, ChatGPT generated 17 Correct, 6 Incomplete, 0 Incorrect, and 2 Potentially Harmful responses; Claude generated 20 Correct, 3 Incomplete, 1 Incorrect, and 1 Potentially Harmful response; Gemini generated 19 Correct, 1 Incomplete, 4 Incorrect, and 1 Potentially Harmful response (Figure 2). Notably, incorrect responses were identified primarily in Claude and Gemini. Additionally, all three models generated Potentially Harmful content.

### 3.3 Readability analysis

**Figure 3:**
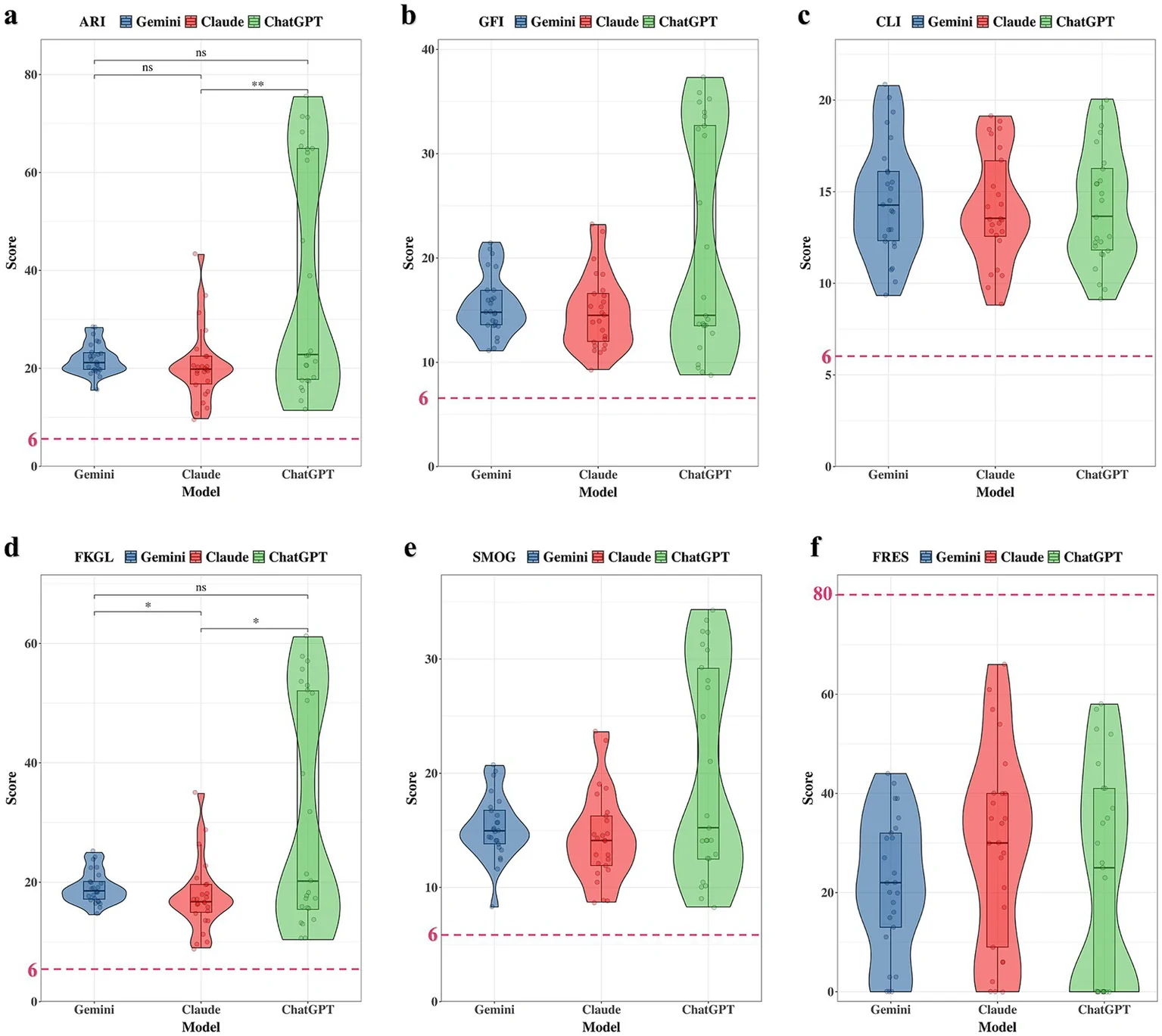
Distribution of readability scores across LLMs. Scores were evaluated using 6 metrics: **(A)** ARI, **(B)** GFI, **(C)** CLI, **(D)** FKGL, **(E)** SMOG, **(F)** FRES. Higher scores indicate greater reading difficulty for ARI/GFI/CLI/FKGL/SMOG, whereas higher FRES indicates better readability. Sixth-grade benchmark values are shown as red reference lines. Statistically significant differences are indicated by asterisks (*p < 0.05, **p < 0.01, NS, not significant).

The readability of the responses generated by the three LLMs was evaluated utilizing six metrics (FRES, ARI, GFI, CLI, FKGL, and SMOG) and compared against the recommended sixth-grade reading benchmark. Significant overall differences were observed only for the ARI (*p* = 0.009) and FKGL (*p* = 0.013) (Table 4 and Figure 3). *Post hoc* pairwise analysis showed that Claude exhibited significantly lower reading difficulty than ChatGPT across both the ARI (*p*_adj = 0.009, *r* = 0.420) and the FKGL (*p*_adj = 0.022, *r* = 0.380). Additionally, Claude demonstrated significantly better readability than Gemini based on the FKGL metric (*p*_adj = 0.049, *r* = 0.340). No significant differences were detected between Gemini and ChatGPT on either ARI or FKGL (Table 5). Notably, the absolute number of responses across all three models that successfully met the recommended sixth-grade reading thresholds was zero. One-sample Wilcoxon signed-rank tests further confirmed that readability scores for all models across all metrics significantly deviated from the grade 6 benchmark (*p*_adj < 0.001). The complete test statistics are detailed in Supplementary Table S8.

**Table 4:** Readability scores [median (Q1, Q3)] of LLM-generated information and comparisons with the sixth-grade benchmark.

Model	FRES	ARI	GFI	CLI	FKGL	SMOG
Gemini 3.5 Flash	22.00 (12.00, 32.5)	21.18 (19.74, 23.89)	14.80 (13.55, 16.90)	14.27 (12.28, 16.45)	18.56 (17.06, 20.70)	14.96 (13.67, 16.88)
Claude Opus 4.8	30.00 (7.50, 40.00)	19.84 (16.07, 22.52)	14.50 (11.85, 16.75)	13.55 (12.46, 17.09)	16.73 (14.32, 19.68)	14.11 (11.75, 16.43)
ChatGPT 5.5	25.00 (0.00, 41.00)	22.83 (17.52, 64.99)	14.50 (13.15, 33.10)	13.66 (11.68, 16.44)	20.19 (15.46, 52.58)	15.23 (12.48, 30.01)
*P*	0.110	0.009*	0.326	0.887	0.013*	0.074
Grade 6 level score	80–90	6	6	6	6	6

*Statistical significance was set at p < 0.05. One-sample Wilcoxon signed-rank tests revealed that readability scores for all three models across all metrics significantly deviated from the grade 6 benchmark (all *p*_adj < 0.001).

**Table 5:** Post hoc pairwise comparisons of readability scores using Wilcoxon signed-rank tests (Bonferroni-adjusted *p-*values).

Comparison	ARI			FKGL		
	*P*_adj	*r*	Median difference (95% CI)	*P*_adj	*r*	Median difference (95% CI)
Gemini 3.5 Flash – Claude Opus 4.8	0.102	0.300	1.910 (−0.060, 3.845)	0.049*	0.340	1.890 (0.370, 3.515)
Claude Opus 4.8 – ChatGPT 5.5	0.009*	0.420	−17.900 (−25.470, −5.105)	0.022*	0.380	−14.065 (−19.640, −4.200)
Gemini 3.5 Flash – ChatGPT 5.5	1.000	0.120	−18.510 (−23.585, −0.570)	1.000	0.040	−14.040 (−18.335, 0.255)

*P*_adj represents the *p*-values adjusted via the Bonferroni correction for three pairwise comparisons (*p*_adj = *p* × 3). *Statistical significance was set at *p*_adj < 0.05. *r* is the effect size for Wilcoxon signed-rank test. 95% CI for median difference estimated via Hodges–Lehmann method.

## 4 Discussion

Driven by advancements in digital health technologies, LLMs have increasingly become a crucial channel for patients to acquire health information. This study systematically evaluated the performance of Gemini 3.5 Flash, Claude Opus 4.8, and ChatGPT 5.5 in addressing common MI-related inquiries. The findings indicate notable performance disparities among the models regarding information quality, transparency and readability. Although LLMs can deliver structured information with specific clinical reference value, their textual readability significantly exceeds the comprehension threshold of the general public, and they universally lack transparency regarding information sources. Currently, existing LLMs can only serve as supplementary educational tools, and their inherent limitations restrict their practicality as standalone health education tools.

### 4.1 Quality and transparency of information

In the quality and transparency assessment, four validated tools were utilized to reveal a core characteristic of LLM-generated MI health information: the coexistence of highly structured information presentation and a lack of underlying evidence-based depth. Overall, the models performed exceptionally well in the EQIP and GQS evaluations but exhibited systemic limitations in the DISCERN and JAMA benchmark assessments.

The median DISCERN scores for all three models fell within the “average” range, aligning with prior research findings (32). However, this finding must be interpreted with caution and should not be simplified as a definitive indicator of the models’ generic unreliability. Methodologically, this generalized outcome was inherently driven by our study design, which evaluated all 25 questions across all scales to preserve statistical pairing. When we scrutinized the item-level performance, the models consistently received lower DISCERN ratings on questions concerning definitions and emergency actions. This descriptive drop reflects the limited suitability of the DISCERN instrument for non-treatment content rather than genuine unreliability of the language models. In contrast, for explicit therapeutic queries, the models demonstrated a more robust baseline of information depth. Furthermore, the JAMA benchmark scores exposed the vulnerabilities of LLMs regarding information source transparency. When outputting MI diagnosis and treatment recommendations, the models almost entirely failed to provide clear authorship, conflict of interest statements, content update dates, or traceable references. This lack of data traceability has been repeatedly corroborated in previous health informatics evaluations (33, 34), underscoring the technical deficiencies of LLMs, which may stem from inherent flaws in platform level citation and content design, rather than directly indicating that the information is less reliable.

Notably, these LLMs achieved favorable evaluations in the EQIP and GQS scorings. The EQIP and GQS primarily focus on assessing the usability, structural organization, and logical coherence of information. The median EQIP scores for the content generated by Gemini, Claude, and ChatGPT all fell within the “good” category, and their GQS scores were universally classified as “good” or “excellent.” This impressive performance is attributed to the robust natural language orchestration capabilities of LLMs, enabling them to package fragmented medical knowledge into highly articulate and logically coherent outputs. However, it is crucial to remain vigilant, as this proficiency sometimes creates a “fluency illusion” (35, 36). Consequently, patients lacking a medical background might instinctively judge the professionalism of the information based on structural formatting and textual fluency. In such instances, the outstanding performance of LLMs on the EQIP and GQS frequently masks critical inaccuracies and high-risk directives, thereby misleading patients into placing undue trust in incomplete or incorrect treatment recommendations, which may result in delayed medical intervention or even patient harm.

### 4.2 Clinical accuracy and patient safety risks

The clinical accuracy and safety assessment revealed a critical gap between the structural quality of LLM-generated responses and their substantive clinical reliability. Despite achieving high scores on these metrics, all three models generated Potentially Harmful content specifically during emergency first-aid scenarios. The algorithms exhibited a pronounced tendency to over-generate content in life-or-death emergency scenarios, injecting generic, non-cardiac first-aid instructions (such as managing severe bleeding, immobilizing spinal trauma, applying the Heimlich maneuver, and utilizing EpiPens for anaphylaxis) into the specific context of acute myocardial infarction. For a layperson experiencing an acute coronary event, encountering these irrelevant and potentially harmful instructions could fatally distract from the singular priority of strict absolute rest and immediate emergency medical services activation. In the context of suspected acute myocardial infarction, these irrelevant instructions represent critical information pollutants that could dangerously delay reperfusion therapy.

Furthermore, Incorrect responses were identified primarily in Claude and Gemini due to mechanistic hallucinations. Specifically, aspirin was repeatedly and erroneously described as having the capacity to “dissolve” or “break down” a blood clot, perilously confounding its antiplatelet function with thrombolytic therapy. Incomplete responses generally involved omitted guideline-directed medical therapies (such as presenting only a single 81 mg aspirin option for long-term care without mentioning essential dual antiplatelet therapy, or failing to establish the differentiating role of high-sensitivity troponin assays between angina and myocardial infarction). While LLMs demonstrate promise in organizing general health education materials, they inevitably produce some incomplete, incorrect, or even potentially harmful responses. In conclusion, current LLMs are limited to being preliminary educational tools for basic MI concepts. Lacking domain-specific safeguards, data transparency, and the ability to evaluate complex treatments, their safety as independent tools for acute management or emergency triage is unsubstantiated. Clinicians utilizing them for patient education must explicitly warn patients about the risks of misinformation.

### 4.3 Readability of information

Readability is a critical determinant of whether health information can be effectively comprehended and utilized by the general public. In this study, the textual readability of LLMs responding to common MI queries was systematically evaluated utilizing six authoritative readability assessment tools. However, some observed readability scores must be interpreted with caution, as they are partially influenced by the structural formatting of the responses. For example, some ChatGPT-generated texts employed semicolons to concatenate multiple independent clauses into a single grammatically valid sentence, which could inflate the values of ARI and FKGL. Nevertheless, the SMOG that relies solely on lexical complexity also scored significantly higher than the benchmark, and none of the content generated by the three models met the recommended sixth-grade reading level. This suggests that lexical complexity and information density may have created a greater burden on readability. While these models are indeed capable of generating detailed and clear clinical information, this is achieved at the expense of textual readability; the content predominantly necessitates high school or college-level reading proficiency for effective comprehension. Regardless of the underlying mechanisms, the generated responses frequently utilized complex syntax and multisyllabic medical terminology. While this preserves the formal rigor of medical information, it intrinsically compromises audience comprehension. When informational reading thresholds are high and devoid of layperson explanations, the content cannot be effectively deciphered by populations with lower health literacy. Given that previous studies associate lower health literacy with poorer cardiovascular outcomes (37–39), the high reading difficulty of LLM outputs highlights a barrier to effective digital health communication, posing a theoretical risk to equitable patient education.

Notably, this phenomenon of suboptimal readability is not exclusive to LLMs but represents a ubiquitous challenge within digital health communication. Prior readability evaluations of cardiovascular disease-related information on the traditional Internet have similarly revealed that the reading difficulty of the majority of online educational materials far exceeds the average comprehension level of the general public (40, 41). Furthermore, several health education evaluations of LLMs in endocrinology and orthopedics have yielded analogous conclusions, indicating that the model-generated texts struggle to satisfy the readability standards required for patient educational materials (42–45). In summary, although current LLMs can generate structurally clear MI-related information possessing certain clinical reference value, the text universally exhibits profound readability deficits. This severely restricts their applicability as patient educational tools, particularly for populations with lower health literacy. Future model optimization must integrate readability enhancements into the training objectives while ensuring information quality. For instance, specific prompt engineering could be leveraged to constrain the output complexity of LLMs, mandating the use of more concise syntax and the provision of everyday analogical explanations for professional terminology. Alternatively, instruction tuning targeted at low health literacy populations could be conducted to dismantle overly specialized terminological barriers at the algorithmic level, thereby rendering the generated content more congruent with the pragmatic requirements of public health communication.

### 4.4 Limitations

This study has several limitations. First and foremost, the evaluation was inherently constrained by the simplistic prompting strategy employed. To simulate typical real-world patient search behaviors, we utilized zero-shot prompts without specifying any readability constraints. Had the LLMs been explicitly prompted to generate responses tailored to a sixth-grade reading level recommended by the NIH and AMA, the readability metrics of the outputs would likely have improved to a more appropriate level. Consequently, our current findings strictly represent the baseline, uninstructed performance of these models in naturalistic user settings, rather than their optimized potential under expert prompt engineering. Future studies should systematically investigate the efficacy of various readability-oriented prompt strategies in bridging this gap and improving the practical utility of LLM-generated health information. Second, interactions with the commercially deployed LLMs were conducted via their official web interfaces; consequently, generation parameters such as temperature and top-p were governed by default platform settings, precluding the complete standardization of the generation process. Concurrently, these models are subject to temporal constraints; their output content may fluctuate over time due to algorithmic upgrades, policy modifications, or official iterations. Therefore, the present findings exclusively represent performance within a specific temporal window. Third, this investigation was restricted to English plaintext evaluations based on global Google Trends data. As health information-seeking behavior and search query patterns vary substantially across countries, cultures, and languages, our findings may not be generalizable to non-English-speaking populations or to region-specific MI education contexts. Fourth, although a double-blind independent scoring and consensus adjudication mechanism was employed to minimize bias, quality and transparency ratings based on instruments such as the DISCERN, EQIP, GQS, and JAMA inevitably entail a degree of subjective judgment. In addition, there is an inherent conceptual limitation in applying legacy quality assessment instruments to LLMs. For example, the JAMA scores observed in our study partially reflect platform-level citation constraints and conversational content design, rather than definitively indicating low informational reliability. Similarly, since the DISCERN tool focuses on treatment options, its scores may not fully reflect the quality of responses generated by LLMs. And conventional readability formulas are sensitive to sentence segmentation and may be influenced by semicolon-delimited structures in LLM outputs. The development of LLM readability instruments remains an unmet need. Future evaluations should prioritize deploying or developing novel instruments specifically validated for LLM-generated health responses. Finally, the primary focus of this evaluation was the objective quality and readability of the information content itself; the extent to which this information impacts patient health literacy, self-management behaviors, and disease prognosis remains unknown. Future research should be dedicated to conducting prospective real-world studies to evaluate patients’ actual comprehension and clinical benefits following LLM-assisted education, thereby promoting the broader application of LLMs in health education.

## 5 Conclusion

In conclusion, based strictly on the evaluation of single-turn, zero-shot English queries conducted during our study period, current LLMs exhibit significant readability barriers and clinical safety limitations. Although they can generate logically coherent foundational content, they occasionally produce clinically inappropriate directives and their texts vastly exceed the recommended sixth-grade reading comprehension standard. These findings indicate that current LLMs are not yet capable as standalone health educational tools for MI. Future model development must focus on dismantling professional terminology barriers and building clinical safety guardrails before such tools can be safely generalized in public health education.

## Data Availability

The original contributions presented in the study are included in the article/Supplementary material, further inquiries can be directed to the corresponding author/s.
