# Supplementary material for: Evaluating large language models for myocardial infarction public health education: a comparative study on information quality, transparency and readability
Source: Front Public Health. 2026 Sep 10;14:1914167. doi: 10.3389/fpubh.2026.1914167 (PMC13601339; doi:10.3389/fpubh.2026.1914167)
Supplement: Supplementary file 1 [file Table_1.DOCX]

**Supplementary Table S1 Google Trends data of the 25 most significant keywords queried globally for myocardial Infarction between 2021–2026.**

| TOP | Query |
| --- | --- |
| 1 | acute myocardial infarction |
| 2 | what is myocardial infarction |
| 3 | what is a myocardial infarction |
| 4 | myocardial infarction symptoms |
| 5 | infarction meaning |
| 6 | myocardial infarction meaning |
| 7 | mi |
| 8 | mi myocardial infarction |
| 9 | ecg |
| 10 | myocardial infarction ecg |
| 11 | infarction definition |
| 12 | myocardial infarction definition |
| 13 | angina |
| 14 | myocardial infarction treatment |
| 15 | heart failure |
| 16 | heart attack |
| 17 | myocardial infarction pdf |
| 18 | myocardial infarction causes |
| 19 | stroke |
| 20 | heart disease |
| 21 | myocardial infarction management |
| 22 | st elevation |
| 23 | icd 10 myocardial infarction |
| 24 | symptoms of myocardial infarction |
| 25 | st elevation myocardial infarction |

**Supplementary Table S2 Google Trends data of the 25 most significant keywords queried globally for heart attack between 2021–2026.**

| TOP | Query |
| --- | --- |
| 1 | heart attack symptoms |
| 2 | heart attack pain |
| 3 | heart pain |
| 4 | heart attack signs |
| 5 | what is heart attack |
| 6 | signs of heart attack |
| 7 | heart attack women |
| 8 | symptoms of heart attack |
| 9 | what is a heart attack |
| 10 | heart attack in women |
| 11 | chest pain |
| 12 | signs of a heart attack |
| 13 | stroke |
| 14 | women heart attack symptoms |
| 15 | heart attack heart rate |
| 16 | heart attack blood pressure |
| 17 | heart attack causes |
| 18 | blood pressure |
| 19 | symptoms of a heart attack |
| 20 | heart attack symptoms in women |
| 21 | cardiac arrest |
| 22 | heart attack signs women |
| 23 | heart attack meaning |
| 24 | cause of heart attack |
| 25 | heart attack lyrics |

**Supplementary Table S3 Mapping and Transformation Matrix from Raw Google Trends Queries to Finalized Patient-Oriented Questions.**

| Final Question No. | Final Patient-Oriented Question (from Table 1) | Corresponding Original Google Query (from S1/S2) |
| --- | --- | --- |
| 1 | What is a heart attack (myocardial infarction)? | acute myocardial infarction; what is myocardial infarction; heart attack |
| 2 | How does a heart attack happen? | infarction meaning; myocardial infarction definition |
| 3 | What is the difference between a heart attack and angina? | angina |
| 4 | What are the common causes and risk factors for a heart attack? | myocardial infarction causes; heart attack causes |
| 5 | Are heart attacks hereditary? | —**Expert-supplemented** |
| 6 | Is there a link between a heart attack and a stroke? | stroke |
| 7 | What is the most typical symptom during a heart attack? | myocardial infarction symptoms; heart attack symptoms; chest pain |
| 8 | How long does the chest pain usually last during a heart attack? | Disaggregated from "myocardial infarction symptoms" |
| 9 | Besides chest pain, what are the less common symptoms of a heart attack? | Disaggregated from "myocardial infarction symptoms" |
| 10 | How do heart attack symptoms differ between men and women? | heart attack women; heart attack symptoms in women |
| 11 | If I or someone near me suddenly has symptoms of a heart attack, what should I do first? | —**Expert-supplemented** |
| 12 | While waiting for an ambulance, what first-aid measures can a family member or bystander take? | —**Expert-supplemented** |
| 13 | If I am alone and suspect a heart attack, what are the safest steps to take? | —**Expert-supplemented** |
| 14 | What tests do doctors usually perform to diagnose a heart attack? | ECG; myocardial infarction ECG |
| 15 | What does an electrocardiogram tell the doctor during a heart attack? | Disaggregated from "ECG / myocardial infarction ECG" |
| 16 | What are the main treatments for a heart attack? | myocardial infarction treatment |
| 17 | What is an emergency stent procedure, and how does it open a blocked artery? | Disaggregated from "myocardial infarction treatment" |
| 18 | Which patients who have had a heart attack need to consider bypass surgery? | Disaggregated from "myocardial infarction treatment" |
| 19 | For patients diagnosed with CAD, how can a heart attack be prevented? | myocardial infarction management |
| 20 | Why is long-term medication still necessary after recovering from a heart attack? | —**Expert-supplemented** |
| 21 | What are the essential long-term medications after a heart attack, and what does each one do? | —**Expert-supplemented** |
| 22 | What is cardiac rehabilitation, and what are its benefits for recovery after a heart attack? | —**Expert-supplemented** |
| 23 | After discharge, what lifestyle changes can help prevent another heart attack? | Disaggregated from "myocardial infarction management" |
| 24 | What dietary changes are important after a heart attack? | —**Expert-supplemented** |
| 25 | After recovering, when can patients return to work, exercise, and sexual activity? | —**Expert-supplemented** |

**Supplementary Table S5 Readability tools, formulas and descriptions.**

| Readability index | Description | Formula |
| --- | --- | --- |
| Flesch Reading Ease Score (FRES) | It was created to assess the readability of newspapers and is particularly effective for evaluating school textbooks and technical manuals. This standardized test is utilized by numerous US government agencies. The scores range from 0 to 100, with higher scores indicating greater ease of reading. | I = (206.835 − (84.6 × (B/W)) − (1.015 × (W/S))) |
| Gunning Fog Index (GFI) | It was designed to assist American businesses in enhancing the readability of their written content and is applicable across various disciplines. It estimates the number of years of education required for a person to understand a given text. | G = 0.4 × (W/S+((C*/W) × 100)) |
| Simple Measure of Gobbledygook (SMOG) | It is typically appropriate for middle-aged readers, ranging from 4th grade to college level. While it aims to test 100% comprehension, most formulas measure about 50%-75% comprehension. It is most accurate when applied to documents that are at least 30 sentences long. It measures the number of years of education the average person needs to understand a text. | G = 1.0430 × √C + 3.1291 |
| Flesch–Kincaid Grade Level (FKGL) | Part of the Kincaid Navy Personnel test collection, it was designed for technical documentation and is suitable for a wide range of disciplines. Delineates the academic capacity level imperative for grasping the written material | G = (11.8 × (B/W)) + (0.39 × (W/S)) − 15.59 |
| Automated readability Index (ARI) | The ARI (Automated Readability Index) has been utilized by the military for writing technical manuals. Its calculation provides the grade level required to comprehend the text. Assesses the scholastic rank in American educational institutions needed to be capable of comprehending written material. The greater the number of characters, the more complex the term. | G = 4.71 × l + 0.5 × ASL − 21.43 |
| Coleman–Liau Index (CLI) | It is designed for middle-aged readers, spanning from 4th grade to college level. The formula is based on text with a grade level range of 0.4 to 16.3 and is applicable to many industries. Evaluates the educational level required for understanding a text and offers an associated grade level in the US education system. | G = (−27.4004 × (E/100)) + 23.06395 |

ASL = the average number of sentences per 100 words, B = number of syllables, C = complex words (≥3 syllables), C* = complex words with exceptions including, proper nouns, words made 3 syllables by addition of “ed” or “es,” compound words made of simpler words, E = predicted Cloze percentage = 141.8401 − (0.214590 × number of characters) + (1.079812 × S), G = grade level, I = Flesch Index Score, S = number of sentences, W = number of words.

**Supplementary Table S8 One-sample Wilcoxon signed-rank test results for readability threshold comparisons (Bonferroni-corrected).**

| **Readability Metric** **(Benchmark)** | **Model** | **Target Met n (%)** | ***Z*-statistic** | ***P*_adj** | ***r*** | **Median Difference (95% CI)** |
| --- | --- | --- | --- | --- | --- | --- |
| FRES  (Target≥80) | Gemini 3.5 Flash | 0/25 (0%) | -4.37 | < 0.001 | 0.87 | -58.03 (-64.00, -52.50) |
|  | Claude Opus 4.8 | 0/25 (0%) | -4.37 | < 0.001 | 0.87 | -50.50 (-60.00, -42.50) |
|  | ChatGPT 5.5 | 0/25 (0%) | -4.42 | < 0.001 | 0.88 | -59.50 (-67.00, -50.00) |
| ARI  (Target≤6) | Gemini 3.5 Flash | 0/25 (0%) | -4.37 | < 0.001 | 0.87 | 15.72 (14.48, 17.30) |
|  | Claude Opus 4.8 | 0/25 (0%) | -4.37 | < 0.001 | 0.87 | 13.78 (11.34, 16.41) |
|  | ChatGPT 5.5 | 0/25 (0%) | -4.37 | < 0.001 | 0.88 | 34.96 (15.45, 39.50) |
| GFI  (Target≤6) | Gemini 3.5 Flash | 0/25 (0%) | -4.37 | < 0.001 | 0.87 | 9.25 (8.10, 10.60) |
|  | Claude Opus 4.8 | 0/25 (0%) | -4.37 | < 0.001 | 0.87 | 8.60 (7.20, 10.20) |
|  | ChatGPT 5.5 | 0/25 (0%) | -4.37 | < 0.001 | 0.87 | 16.25 (8.00, 18.50) |
| CLI  (Target≤6) | Gemini 3.5 Flash | 0/25 (0%) | -4.37 | < 0.001 | 0.87 | 8.48 (7.12, 9.84) |
|  | Claude Opus 4.8 | 0/25 (0%) | -4.37 | < 0.001 | 0.87 | 7.98 (6.84, 9.60) |
|  | ChatGPT 5.5 | 0/25 (0%) | -4.37 | < 0.001 | 0.87 | 7.97 (6.51, 9.46) |
| FKGL  (Target≤6) | Gemini 3.5 Flash | 0/25 (0%) | -4.37 | < 0.001 | 0.87 | 12.83 (11.81, 14.31) |
|  | Claude Opus 4.8 | 0/25 (0%) | -4.37 | < 0.001 | 0.87 | 10.96 (9.16, 13.19) |
|  | ChatGPT 5.5 | 0/25 (0%) | -4.37 | < 0.001 | 0.87 | 27.51 (12.16, 31.14) |
| SMOG  (Target≤6) | Gemini 3.5 Flash | 0/25 (0%) | -4.37 | < 0.001 | 0.87 | 9.08 (8.12, 10.33) |
|  | Claude Opus 4.8 | 0/25 (0%) | -4.37 | < 0.001 | 0.87 | 8.11 (6.60, 9.83) |
|  | ChatGPT 5.5 | 0/25 (0%) | -4.37 | < 0.001 | 0.87 | 14.64 (8.11, 17.24) |

*P*_adj represents p-values strictly adjusted via the Bonferroni correction for 18 multiple comparisons (*p*×18). *r* is the effect size for Wilcoxon signed-rank test. A negative median difference for FRES indicates scores below the benchmark (>80); a positive median difference for other metrics indicates scores above the benchmark (<6). 95% Confidence Intervals (CIs) for the median differences were estimated via the exact Hodges-Lehmann method.
